# Clinical Lecture on Gonorrhœa and Imaginary Spermatorrhœa

**Published:** 1864-09

**Authors:** Thomas K. Chambers


ART. IV.—*Clinical Lecture on Gonorrhœa and Imaginary Spermatorrhœa.* Given at St. Mary's Hospital, June 14th, 1861. By THOMAS K. CHAMBERS, M. D., etc.

The poison of gonorrhœa, as a rule, attaches itself to the urethra alone, and (like all animal poisons on mucous membranes) has a tendency to run a definite course, to exhaust its virulence by the formation of pus, and so to cure itself. I always, therefore, leave these hospital cases quite alone for your instruction; and, if they are recent, you see them get quite well of their own accord; sometimes in four or five days, as happened in the case before us; sometimes after a longer period; but always without any unfavorable symptoms. The fact is that gonorrhœa is naturally, in both male and female, a most mild disease, with power to get well in about a fortnight by the simplest treatment, if only it is not made severe by the folly of the patient or his medical attendant. I consider all primary heroic treatment of urethral discharges a most unjustifiable interference with nature.

Doubtless a good many patients are tough enough to bear without ill consequences local tampering with uretha; but very frequently you will have stricture and swelled testicle follow, and every now and then you will have a result which will weigh on your conscience for the rest of your lives. You may have the blood of a fellow creature on your head.

Capable as the urinary mucous membrane is of taking care of itself and getting rid of poisons which are in a manner natural to it, it resents foreign irritants; and it resents them most particularly when it is not guarded by a purulent coating and is in a healthy state.

Real spermatorrhœa is a most rare, almost unknown disease. If you ask all the physicians and surgeons of prominent standing, the hospital staffs in this metropolis, they will tell you they are doubtful if they ever saw a decided case. But of *imaginary* spermatorrhœa they have almost daily instances in private practice. Persons whose minds are unhinged with the horrid suggestion are constantly coming before them; and if the poor creatures will confess to the source of their impression, it may generally be traced to the reading of beastly books or advertisements in the newspapers. You could scarcely imagine how enormous is the circulation of these publications; under specious titles, they are advertized at the cost of about £20,000 a year, and are sent all over the globe. When traveling with the Prince of Wales two years ago, I found them wherever I found Englishmen; and a captain in Her Majesty's navy told me he had caught them in the hands of his midshipmen in the Mediterranean.

The nature of the cases under the delusions of these ingenious traps, and presenting themselves to you as spermatorrhœa, are various. There are:

1. A gleety discharge from slight stricture.
2. A similar discharge from pure debility, exactly analogous to leucorrhœa in the female. This is common in the active minded, who use much mental exertion at night, such as students cramming for examination, etc.
3. Incapacity to complete the generative act from mental agitation. This happens to new-married men from modest respect, and to the unmarried from consciousness of sin, and disgust towards the female. The same mental agitation produces simultaneously indigestion and a deposit of lithates in the urine, which is then supposed to be semen.
4. Nocturnal pollutions, arising solely from the habit of oversleeping; but rarely affecting the health, except secondarily through the mind.
5. Slight epileptic fits.
6. Simple delusions, grounded on no symptom, but taking this form on account of the secret publicity given to the gross exaggerations and falsehoods contained in the books I have alluded to.
7. The fallacies which harass these poor people are often the more deeply ingrained by the consciousness of dirty habits in their boyhood, though probably those habits have been long left off, and have never been practiced to the extent of injuring the health. But sometimes you will find patients complaining of "spermatorrhœa," half with the idea of deceiving, and half as a euphemistic way of telling you that they are still addicted to self-pollution.

